# Reference Ranges of Amniotic Fluid Index in Late Third Trimester of Pregnancy: What Should the Optimal Interval between Two Ultrasound Examinations Be?

**DOI:** 10.1155/2015/319204

**Published:** 2015-01-15

**Authors:** Shripad Hebbar, Lavanya Rai, Prashant Adiga, Shyamala Guruvare

**Affiliations:** Department of Obstetrics and Gynaecology, Kasturba Medical College, Manipal University, Manipal 576 104, India

## Abstract

*Background*. Amniotic fluid index (AFI) is one of the major and deciding components of fetal biophysical profile and by itself it can predict pregnancy outcome. Very low values are associated with intrauterine growth restriction and renal anomalies of fetus, whereas high values may indicate fetal GI anomalies, maternal diabetes mellitus, and so forth. However, before deciding the cut-off standards for abnormal values for a local population, what constitutes a normal range for specific gestational age and the ideal interval of testing should be defined. *Objectives*. To establish reference standards for AFI for local population after 34 weeks of pregnancy and to decide an optimal scan interval for AFI estimation in third trimester in low risk antenatal women. *Materials and Methods*. A prospective estimation of AFI was done in 50 healthy pregnant women from 34 to 40 weeks at weekly intervals. The trend of amniotic fluid volume was studied with advancing gestational age. Only low risk singleton pregnancies with accurately established gestational age who were available for all weekly scan from 34 to 40 weeks were included in the study. Women with gestational or overt diabetes mellitus, hypertensive disorders of the pregnancy, prelabour rupture of membranes, and congenital anomalies in the foetus and those who delivered before 40 completed weeks were excluded from the study. For the purpose of AFI measurement, the uterine cavity was arbitrarily divided into four quadrants by a vertical and horizontal line running through umbilicus. Linear array transabdominal probe was used to measure the largest vertical pocket (in cm) in perpendicular plane to the abdominal skin in each quadrant. Amniotic fluid index was obtained by adding these four measurements. Statistical analysis was done using SPSS software (Version 16, Chicago, IL). Percentile curves (5th, 50th, and 95th centiles) were constructed for comparison with other studies. Cohen's *d* coefficient was used to examine the magnitude of change at different time intervals. *Results*. Starting from 34 weeks till 40 weeks, 50 ultrasound measurements were available at each gestational age. The mean (standard deviation) of AFI values (in cms) were 34 W: 14.59 (1.79), 35 W: 14.25 (1.57), 36 W: 13.17 (1.56), 37 W: 12.48 (1.52), 38 W: 12.2 (1.7), and 39 W: 11.37 (1.71). The 5th percentile cut-off was 8.7 cm at 40 weeks. There was a gradual decline of AFI values as the gestational age approached term. Significant drop in AFI was noted at two-week intervals. AFI curve generated from the study varied significantly when compared with already published data, both from India and abroad. *Conclusion*. Normative range for AFI values for late third trimester was established. Appreciable changes occurred in AFI values as gestation advanced by two weeks. Hence, it is recommended to follow up low risk antenatal women every two weeks after 34 weeks of pregnancy. The percentile curves of AFI obtained from the present study may be used to detect abnormalities of amniotic fluid for our population.

## 1. Introduction

The ultimate goal of antepartum surveillance program is to improve perinatal outcome and to decrease intrauterine fetal demise besides prevention of maternal morbidity and mortality [1, 2]. A fetus in distress should be identified at the earliest so that timely delivery will not only salvage the fetus but also prevent long term neurological impairments such as injury to fetal central nervous system [3]. Though it is said that such an event is more common in high risk pregnancies, the fetuses belonging to low risk mothers are not totally immune [4]. There are definite guidelines for frequency of antenatal testing for high risk pregnant women, but what constitutes an ideal screening program for low risk pregnancies is still unknown [5].

Amniotic fluid assessment by ultrasound is one of the important tools in assessing the fetal health in all risk categories especially beyond the period of viability [6]. Though there are several ways [7] to assess quantity of amniotic fluid ranging from clinical palpation to measurement of single deepest vertical pocket [8], amniotic fluid index (AFI) by four-quadrant technique as described by Phelan et al. [9] in 1987 and among them AFI is popular and reliable method of quantifying amniotic fluid till today. AFI is one of the essential components of fetal biophysical profile (BPP) and its values correlate well with adequacy of fetal renal perfusion. Normally it peaks at 32 to 34 weeks of gestation and thereafter there is a gradual reduction in amniotic fluid due to increase in concentrating capacity of fetal kidneys [10]. However, a drastic reduction in its quantity may indicate underlying placental insufficiency, which has definite implications on growing fetus. The values between 8 and 25 are considered to be normal, 5–8 low normal, and less than 5 oligoamnios [11]. At values less than 5, there is higher incidence of perinatal morbidity and mortality and many a time immediate delivery is the only way out [12, 13]. Hence it is very important to scan the patient to note such a trend periodically during antenatal visits. AFI is the fifth parameter in traditional five-point biophysical profile and second parameter in rapid two-point modified BPP (the other one being NST) [14]. Though there is no definite said protocol for identifying compromised fetus, many believe that biweekly nonstress test and AFI assessment should be offered to all women at risk [15]. But what constitutes an ideal frequency of AFI monitoring for low risk pregnancy is still unknown. Frequent monitoring adds to the cost and maternal anxiety and optimizing the ultrasound examinations is the need of the day.

The present study is an effort to examine the quantum of decrease in AFI in the third trimester and interval of scanning to detect a significant change, thereby formulating guidelines for antenatal ultrasound examinations in low risk women.

## 2. Aims and Objectives

The purpose of the present investigation is

1. to study the pattern of change in AFI on weekly basis from 34 weeks till delivery;
2. to constitute reference ranges of AFI from 34 to 40 weeks of gestation;
3. to find the time interval by which there is a significant fall in AFI, which will help obstetrician to plan an ideal protocol for antenatal ultrasound examination in the third trimester.

## 3. Materials and Methods

This was a prospective observational study conducted at the Department of Obstetrics and Gynaecology, Kasturba Medical College, Manipal, from January 2012 to December 2012. Institutional ethical committee approval was obtained prior to study. Inclusion criteria were low risk singleton pregnancy, starting gestational age of 34 weeks, reliable last menstrual period and dates correlated and confirmed by comparison with first trimester CRL (Crown Rump Length). Once initial criteria were met, those who were subsequently diagnosed to have abnormalities of liquor volume due to conditions such as hypertensive disorders, gestational diabetes, and placental insufficiency were excluded from the study, so as to obtain normative data. Only those patients who delivered at 40 weeks were included in the study as we wanted longitudinal data till term. The final study subjects were 50 low risk pregnant women who underwent serial scans at weekly interval starting from 34 weeks till term.

The subjects belonged to the local population consisting mainly of Tuluva, Billava, Bunt, Koraga, Kulala, Devadiga, Konkanis, Shivalli Brahmins, Bayri Muslim, and Catholic communities, the spoken language mainly being Kannada, Tulu, and Konkani. The women were medium built, the average height was of 152 to 156 cms, and prepregnancy weight was between 45 and 50 kg.

The ultrasound examination was carried out after instructing the patient to empty her bladder. The examinations were performed with a convex 3.5 MHz probe (Philips HD11XE ultrasound equipment). The patient was asked to lie down in supine position. Uterus was arbitrarily divided into four quadrants using linea nigra as a vertical line and a transverse line passing through umbilicus, as described by Phelan et al. [9]. The transducer was placed in each of these quadrants in sagittal plane perpendicular to patient's abdomen and maximum depth of amniotic fluid was calculated in centimeters excluding the cord loops and small fetal parts. Caution was exercised to avoid excessive pressure on the transducer as it can alter AFI measurements. The values of all four quadrants were added to obtain the final amniotic fluid index (AFI).

### 3.1. Sample Size Estimation

Khadilkar et al. [16] from the Department of Obstetrics and Gynaecology, Grant Medical College, Mumbai, conducted a prospective, cross sectional study in low risk healthy pregnant subjects to obtain a gestational reference range for AFI among Indian women. They noted that the mean and standard deviation of AFI (cm) at 34 weeks of gestation was 14.2 and 2.4, respectively. We hypothesised that a difference of 1.5 cm in the mean AFI would be significantly different from the normal values and accordingly estimated sample size to show a desired level of power of 90% and level of significance 0.05, by using the formula,

(1) $$\begin{array}{c} n={\left(\frac{\left(z\alpha +z\beta \right)\sigma }{\mu 1-\mu 0}\right)}^{2}, \end{array}$$

where *zα* = 1.96 (critical value that divides the central 95% of *z* distribution from 5% in the tails), *zβ* = 1.28 (critical value that separates the lower 10% of distribution from upper 90%), *σ* = standard deviation, and *μ*1 − *μ*0 = difference of two means.

Accordingly it was estimated that 27 patients are required and we decided to recruit 50 patients to have satisfactory results.

## 4. Statistical Methods

Data was analyzed using SPSS version 16 for windows (SPSS Inc., Chicago, IL, USA). Descriptive analysis was performed to obtain mean, standard deviation, and percentile values for AFI from 34 to 40 weeks. Microsoft Excel 2010 was used to plot percentile values (5th, 50th, and 95th) across various gestational ages. A polynomial regression analysis of 3rd order was used to find the best fit. The decline in AFI value was calculated at weekly interval and the magnitude of change was analyzed by effect size estimation (Cohen *d* coefficient) [17].

The formula for Cohen's *d* is given as follows:

(2) $$\begin{array}{c} d=\frac{M_{1}-M_{2}}{\sqrt{\left(s_{1}^{2}+s_{2}^{2}\right)/2}}, \end{array}$$

where *M*_1_ and *M*_2_ are the means and *s*_1_ and *s*_2_ are the standard deviations of two groups.

## 5. Results

Of the 50 patients who were recruited for the study and were between the age of 22 to 28 years, more than half (32 patients, 64%) were primigravidae and 18 (36%) were multigravidae. None of them had any antenatal complications. All of them delivered at around 39+ to 40 weeks. 16 (32%) patients required caesarean delivery for obstetric indication such as failed induction, cephalopelvic disproportion, and fetal distress in labour. The mean (standard deviation) birth weight of the neonates (measured in kg) was 2.83 (0.34), with 1st minute APGAR score (mean and standard deviation) of 8.48 (1.09) and 5th minute APGAR was 8.72 (1.01). As mentioned in methodology, we have excluded those who delivered before term as we required AFI from 34 weeks to 40 weeks of gestation for analysis purpose.

**Figure 1:**
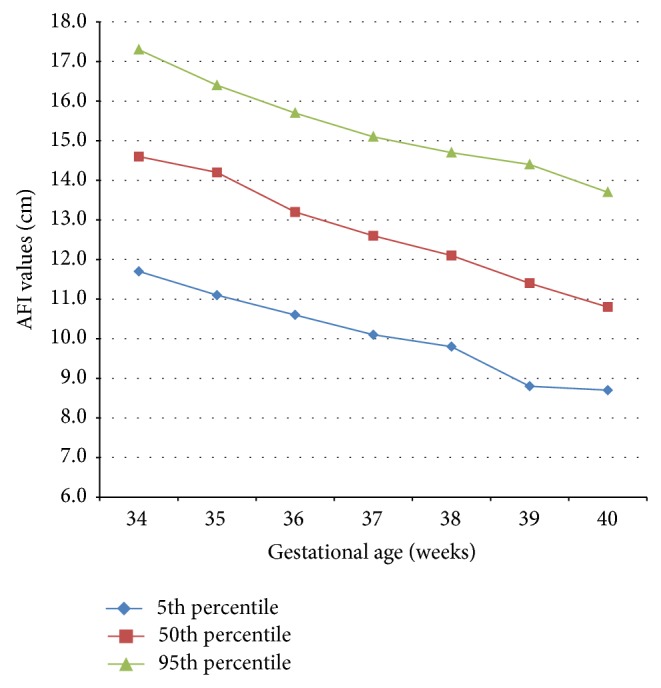
Graphical representation of AFI centiles at various gestational ages.

Table 1 describes the descriptive data for AFI. The AFI values differed throughout the gestation and there was a gradual decline in the values as pregnancy advanced. The 5th, 50th, and 95th percentiles ranged from 11.7, 14.6, and 17.3, respectively, at 34 weeks to 8.7, 10.8, and 13.7, respectively, at 40 weeks. It is interesting to note that all the values were within 8 to 25 cm range (which is accepted and established normal range for AFI values worldwide). The maximum value of AFI in any single patient was 17.6 cm and minimum 8.5 cm in our series of low risk antenatal pregnant women. If minimum (5th centile) and maximum (95th centile) are considered as normal range, it was noted that the corresponding values too were different at different gestational ages; the more advanced the gestational age, the lesser the values. These changes are graphically represented in Figure 1.

We used difference in mean values of one week to the next week to evaluate the decreasing trend of amniotic fluid from 34 to 40 weeks of gestation (Table 2). Dark shaded area indicates cells where calculations are not required as they are the same weeks or previous weeks. It can be seen that many cells have the values less than 1, but still the difference may be calculated statistically significant if ordinary statistical tests such as paired *t* test were applied and hence we have used Cohen's test which very well detects the magnitude of change.

Table 3 indicates Cohen's *d* values for week to week comparison and it can be seen that not much change was seen in immediate week, but changes became significant when the interval between two scans was more than 2 weeks or more in most of the comparisons. Hence from this table there is substantial evidence that liquor volume decreases significantly over the period of 14 days more in low risk antenatal women.

**Figure 2:**
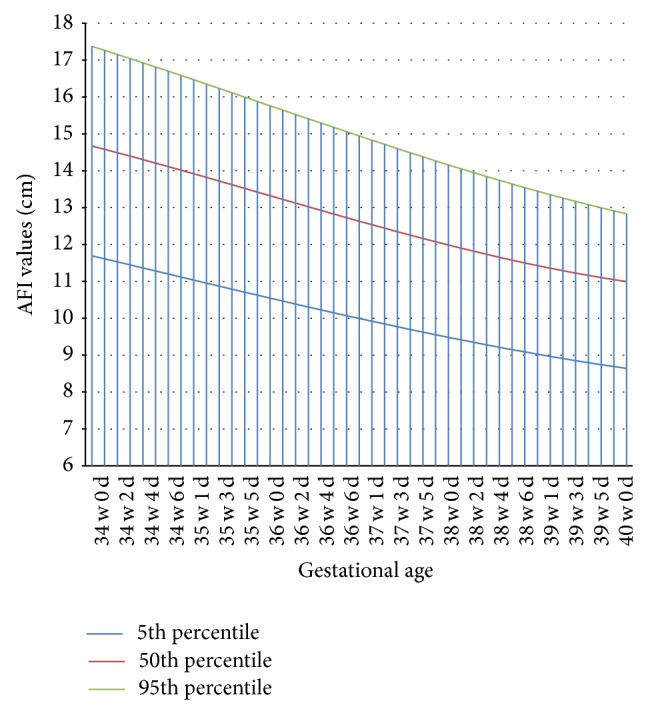
Curve of AFI values (5th, 50th, and 95th centiles) from 34 to 40 weeks following smoothing procedure from polynomial regression of 3rd degree.

Our results indicated that from 34 weeks onwards there is a gradual reduction in AFI. Using polynomial regression analysis, we have established reference standards for AFI ranges from 34 to 40 weeks (Figure 2). The regression analysis further showed that there was a good degree of correlation between GA (gestational age) and AFI (*R* ^2^ = 0.89 to 0.95; *P* < 0.005).

The following equations were derived by third degree polynomial regression using **y** (AFI in cm) as dependent variable and **x** (gestation age in weeks) as independent variable, where *Y* ^5th^, *Y* ^50th^, and *Y* ^95th^ indicate 5th, 50th, and 95th centile values for AFI and GA indicates gestational age in weeks:

(3) Y5th=(−84.8833337026)+(9.46507939511×GA) +(−0.289285715099×GA2) +0.0027777777851×GA3,Y50th=(−283.684761575)+(26.1987698144×GA) +(−0.748571427845×GA2) +0.00694444443791×GA3,Y95th=(−212.166667464)+(20.6884921284×GA) +(−0.598809525566×GA2) +0.00555555557137×GA3.

## 6. Discussion

Amniotic fluid production and regulation is a complex and dynamic process involving the fetus, placenta, and mother. Amniotic fluid volume gradually increases till 32–34 weeks of gestation and thereafter there is a gradual reduction till term [18, 19]. The critical AFI range of 8 to 25 cm signifies fetal well-being and the deviation from this range is associated with increase in fetal and maternal complications due to oligoamnios and polyhydramnios. The third trimester AFI values are proportionate to fetal urine production [20, 21] and hence in normal range indicate good placental perfusion and fetal nutrient and oxygen transfer. Hence monitoring the AFI has become a standard of antenatal care.

**Figure 3:**
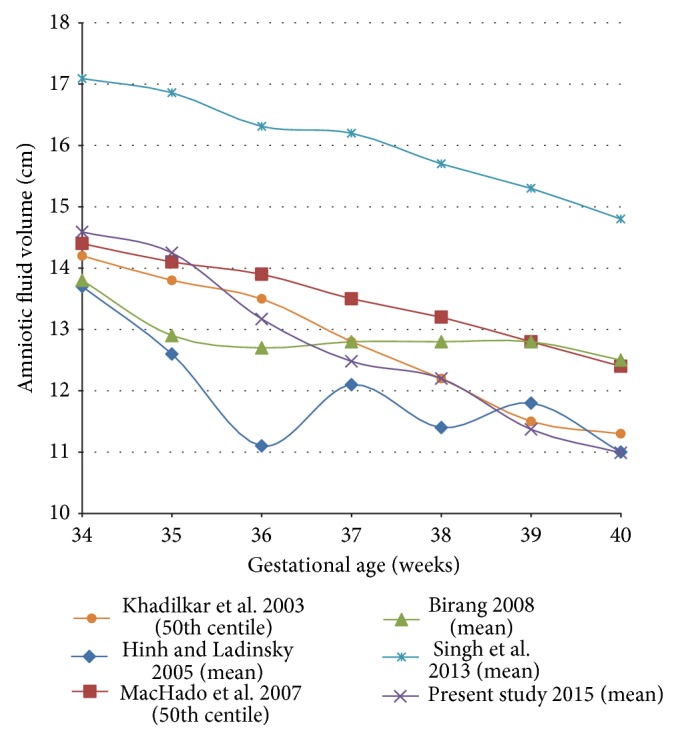
Comparison of AFI values at different gestational ages in various studies.

There is wide variation in reference standards for mean AFI values according to population, race, and geography.  Table 4 compares our finding with that of other authors [16, 22–25]. We have also graphically interpreted findings in the other studies (either mean or 50th percentile values) in Figure 3. However, it is noticeable that majority of the studies agree that from 34 weeks onward there is a gradual fall in AFI values. The two studies [16, 25] are from India, but the reported AFI range has a wide range. This may be because their observations were based upon retrospective cross sectional data. It is noticeable that AFI reference values published by Singh et al. are 2 to 3 cm more than all other series at all gestational ages; we presume this may be because the study was done in Indraprastha Apollo Hospital, New Delhi, where patients from very high socioeconomic status are catered. Khadilkar et al. reported their findings from patients attending antenatal clinic of Grant Medical College, Bombay, and our findings too match with their data. Hence, it can be opined that AFI standards have to be defined for specific populations in order to eliminate bias resulting from socioeconomic groups, geographical locations, race, and so forth. However, it must be noted that almost all authors have reported a steady decline in AFI values with the advancing gestational age, except Birang et al. from Iran. Their series included retrospective cross sectional data and the number differed from minimum of 12 observations at 35 weeks to maximum of 68 observations at 39 weeks. This might be the reason for their finding of rapid fall of AFI from 34 to 35 weeks, plateauing between 37 and 39 weeks and once again slow fall at 40 weeks. Such observations indicate weakness of cross sectional cohort, as the same patients are not followed up sequentially.

Amniotic fluid once thought to be a stagnant pool with approximate turn over time of twenty-four hours. In high risk pregnancies complicated by chronic placental insufficiency liquor is known to drastically reduce in a shorter time and it has been recommended to perform AFI estimation once in three days or at times even frequently depending upon other fetal well-being surveillance tools such as Doppler assessment of fetal circulation. However, there is no universal consensus regarding the frequency of AFI estimation in low risk antenatal women. Hence, it is important to determine a critical interval at which the fall in AFI becomes clinically significant.

We have not used statistical significance test (involving estimation of *P* value) such as* paired t test* for comparing AFI values at different gestational ages, as these tests tend to give significant *P* values even when a minor variation exists in the means of two groups. When sample size is sufficiently large, even the fractional differences are likely to be reported as significant *P* values, hence giving meaningless interpretations. Instead, we have calculated effect size estimate (Cohen *d*) to quantify the changes in the AFI over a period of time.

Effect size is a simple measure for quantifying the difference between two groups or the same group over time, on a common scale. There are several methods mentioned in the literature to calculate the effect sizes (Cohen 1988 [17], Rosenthal and Rosnow 1991 [26], Partial Eta squared Richardson 2011 [27]) and so forth. However, we have used Cohen's *d* estimate as described by Cohen 1988, to calculate effect sizes as this method is easy, simple to understand and can be applied to any measured outcome in scientific study.

From our statistical analysis, we have found that there is no much decrease in AFI at interval of one week, but thereafter the differences become large and significant. Hence, it appears that when the liquor is within normal range, the chances of fetal jeopardy are unlikely to occur within next week; one can safely repeat the AFI after 2 weeks. At the time of estimation of AFI, one can also perform other tests for foetal well-being such as documentation of gross foetal body movements, foetal tone, and foetal breathing movements to be assured that foetus is not hypoxic. In addition, interval biometry may be done at whenever required to quantify satisfactory foetal growth. In the absence of any maternal or foetal risk factors, we are of the opinion that AFI estimation once in fortnight is good enough to ensure satisfactory pregnancy outcome.

## 7. Conclusions

We have established not only gestational specific normative AFI reference standards for late third trimester (34 to 40 weeks) for our local population but also magnitude of change in AFI values at weekly interval by quantitative analysis using effect size statistics. Strength of present study is that it is based on longitudinal data of normal healthy pregnant women and percentile curves obtained can be used to define what constitutes normal range of AFI for low risk antenatal patients. Though our results are based on required number of patients by sample size determination, larger number of subjects if studied may yield robust reference curves for AFI and identify extreme values to define what constitutes oligo- or polyhydramnios. The same study can be extended to high risk pregnancies such as preeclampsia, chronic hypertension, multiple gestation, and intrauterine growth restriction, in order to determine the frequency of liquor testing for these cohorts.

## Figures and Tables

**Table 1:** AFI values from 34 to 40 weeks; mean, standard deviation, and percentile values (all in centimeters).

Gestational age	Mean	Standard deviation	5th percentile	10th percentile	50th percentile	90th percentile	95th percentile
34 weeks	14.59	1.79	11.7	12.0	14.6	17.0	17.3
35 weeks	14.25	1.57	11.1	11.8	14.2	16.2	16.4
36 weeks	13.17	1.56	10.6	11.0	13.2	15.3	15.7
37 weeks	12.48	1.52	10.1	10.2	12.6	14.7	15.1
38 weeks	12.20	1.70	9.8	10.0	12.1	14.4	14.7
39 weeks	11.37	1.71	8.8	9.1	11.4	14.0	14.4
40 weeks	10.99	1.55	8.7	8.8	10.8	13.5	13.7

**Table 2:** Mean change in AFI (cm) values at different intervals.

From	To					
	35 weeks	36 weeks	37 weeks	38 weeks	39 weeks	40 weeks
34 weeks	0.34	1.42	2.12	2.39	3.22	3.61
35 weeks	∗	1.08	1.77	2.05	2.88	3.26
36 weeks	∗	∗	0.7	0.97	1.8	2.19
37 weeks	∗	∗	∗	0.27	1.1	1.49
38 weeks	∗	∗	∗	∗	0.83	1.22
39 weeks	∗	∗	∗	∗	∗	0.39

^*^Comparison not done.

**Table 3:** Cohen *d* coefficients of effect size at different intervals.

From	To					
	35 weeks	36 weeks	37 weeks	38 weeks	39 weeks	40 weeks
34 weeks	0.21	0.85	1.29	1.38	1.86	2.18
35 weeks	#	0.7	1.16	1.27	1.77	2.12
36 weeks	#	#	0.46	0.6	1.11	1.42
37 weeks	#	#	#	0.17	0.69	0.98
38 weeks	#	#	#	#	0.49	0.76
39 weeks	#	#	#	#	#	0.24

0.2–0.49 small effect, 0.5–0.8 medium effect, and >0.8 large effect.

^#^Comparison not done.

**Table 4:** Values of AFI (in cm) by different authors.

Authors	AFI values	34 W	35 W	36 W	37 W	38 W	39 W	40 W
	5th centile	7.6	7.4	7.2	7.0	6.8	6.1	5.9
Khadilkar et al. 2003 [16]	50th centile	14.2	13.8	13.5	12.8	12.2	11.5	11.3
	95th centile	19	18.5	18.3	18.2	17.6	16.8	16.6

	Mean (St. Dev)	13.7 (3.1)	12.6 (2.2)	11.1 (2.6)	12.1 (2.4)	11.4 (2.1)	11.8 (1.7)	11.0 (1.0)
Hinh and Ladinsky 2005 [22]	Min	8.5	8.6	7.1	6.7	6.3	8.4	9.4
	Max	18.8	16.8	16.3	15.9	15.4	14.8	12.7

	10th centile	10.2	9.7	9.1	8.4	7.7	7	6.2
MacHado et al. 2007 [23]	50th centile	14.4	14.1	13.9	13.5	13.2	12.8	12.4
	90th centile	19.5	19.4	19.3	19.1	18.9	18.6	18.3

	Mean (St. Dev)	13.8 (1.18)	12.9 (0.60)	12.7 (1.55)	12.8 (0.84)	12.8 (0.89)	12.8 (1.19)	12.5 (0.98)
Birang 2008 [24]	5th centile	8.3	7.3	7.1	7.1	7.1	7.0	6.6
	95th centile	23.7	23.2	22.8	22.1	20	18.7	18

	Mean	17.1	16.9	16.3	16.2	15.7	15.3	14.8
Singh et al. 2013 [25]	5th centile	11.0	10	9.7	10.1	9.9	8.1	8.8
	95th centile	24.5	24.1	24.8	24.2	24.1	23.7	18

	Mean	14.59 (1.79)	14.25 (1.57)	13.17 (1.56)	12.48 (1.52)	12.2 (1.7)	11.37 (1.71)	10.99 (1.55)
Present study	5th centile	11.7	11.1	10.6	10.1	9.8	8.8	8.7
	95th centile	17.3	16.4	15.7	15.1	14.7	14.4	13.7
